# Scavenger receptor B1, the HDL receptor, is expressed abundantly in liver sinusoidal endothelial cells

**DOI:** 10.1038/srep20646

**Published:** 2016-02-11

**Authors:** Latha P. Ganesan, Jessica M. Mates, Alana M. Cheplowitz, Christina L. Avila, Jason M. Zimmerer, Zhili Yao, Andrei Maiseyeu, Murugesan V. S. Rajaram, John M. Robinson, Clark L. Anderson

**Affiliations:** 1 Department of Internal Medicine, The Ohio State University, Columbus, OH 43210, United States; 2 Department of Surgery, The Ohio State University, Columbus, OH 43210, United States; 3 Department of Medicine, Division of Cardiovascular Medicine, University of Maryland, Baltimore, MD 21201, United States; 4 Department of Microbial Infection and Immunity,The Ohio State University, Columbus, OH 43210, United States; 5 Department of Physiology and Cell Biology, The Ohio State University, Columbus, OH 43210, United States.

## Abstract

Cholesterol from peripheral tissue, carried by HDL, is metabolized in the liver after uptake by the HDL receptor, SR-B1. Hepatocytes have long been considered the only liver cells expressing SR-B1; however, in this study we describe two disparate immunofluorescence (IF) experiments that suggest otherwise. Using high-resolution confocal microscopy employing ultrathin (120 nm) sections of mouse liver, improving z-axis resolution, we identified the liver sinusoidal endothelial cells (LSEC), marked by FcγRIIb, as the cell within the liver expressing abundant SR-B1. In contrast, the hepatocyte, identified with β-catenin, expressed considerably weaker levels, although optical resolution of SR-B1 was inadequate. Thus, we moved to a different IF strategy, first separating dissociated liver cells by gradient centrifugation into two portions, hepatocytes (parenchymal cells) and LSEC (non-parenchymal cells). Characterizing both portions for the cellular expression of SR-B1 by flow cytometry, we found that LSEC expressed considerable amounts of SR-B1 while in hepatocytes SR-B1 expression was barely perceptible. Assessing mRNA of SR-B1 by real time PCR we found messenger expression in LSEC to be about 5 times higher than in hepatocytes.

Cholesterol bound to high density lipoprotein (HDL, C-HDL) somehow lengthens human lifespan, for the higher the concentration in blood, the lower the severity of atherosclerosis^1^. Much research has sought the basis for this remarkable observation. It has been learned that HDL plays a critical role in cholesterol metabolism by facilitating the uptake of excess cholesterol from cells of peripheral organs, transporting cholesterol in blood, and delivering cholesterol to the liver where it is ultimately moved to bile for excretion.

HDL-C in blood engages the liver, it is thought, by interacting with a specific receptor, Scavenger Receptor B1 (SR-B1), which is an 82 kDa integral membrane glycoprotein with a large extracellular domain (403 aa) tethered by two transmembrane domains and short cytoplasmic tails^2^. How SR-B1 binds HDL-C and then unloads cholesterol in the liver is an area of much current work that suggests a variety of mechanisms. It has been proposed, for example, that SR-B1 selectively removes cholesterol from bound HDL-C and internalizes C while leaving C-poor HDL outside the cell, uninternalized^1^,^3^,^4^. Others say that HDL-C particles bound to SR-B1 are endocytosed^5^; cholesterol remains inside the cell while C-poor HDL is exocytosed^6^. Other mechanisms seem possible.

It is known that SR-B1 is abundantly expressed in liver and in other steroidogenic tissues^7^. The site of receptor expression in the liver is reasoned to be the hepatocyte plasma membrane facing the sinusoid^7^,^8^. If indeed SR-B1 is expressed on the sinusoidal domain of the hepatocyte, then, considering liver anatomy, how does HDL-C pass across the liver sinusoidal endothelial cells (LSEC) from blood? The LSEC would appear to act as a hedgerow separating blood circulation from the Space of Disse and the sinusoidal side of hepatocytes, and is generally thought, with no strong supporting data, to act as a sieve, allowing HDL-C to move passively through the fenestrae which occupy 2–20% of the LSEC surface^9^,^10^. However, other features of LSEC need to be considered. These cells are robust scavengers, more so than Kupffer cells, clearing the circulation of small particles such as viruses and small immune complexes^11^,^12^,^13^. They contain abundant coated vesicles and display a variety of endocytic receptors including: scavenger receptors SR-A, Stabilin-1, Stabilin-2^14^,^15^, and receptors for mannose, collagen, hyaluronan, L-SIGN, and Fc receptors (FcγRIIb); but not complement receptors (review)^11^. These cells are built for taking up and remove of plasma constituents; therefore, the LSEC must be more thoroughly evaluated for participation in HDL-C metabolism.

Our interest in this field was stimulated by published IF studies showing SR-B1 localized in the mouse liver to an area of the hepatocyte that, we perceived, was indistinguishable microscopically from the LSEC^16^,^17^. Using two separate high-resolution strategies, IF confocal microscopy of ultrathin cryosections of fixed liver and flow cytometry of isolated liver cells, we have found that the expression of SR-B1 protein in LSEC is markedly higher than the expression of SR-B1 protein within hepatocytes. The implications of these findings are profound for they contradict the current paradigm of HDL-C transit and SR-B1 expression in liver and thereby of cholesterol metabolism.

## Results

### Where in liver is SR-B1 expressed?

The published IF microscopic images of SR-B1 expression in liver are said to show a hepatic sinusoidal pattern^16^,^17^, whereas an identical pattern appears in our published images of RIIb expression in the LSEC, a completely different cell positioned at the sinusoidal border of the hepatocyte^12^. Aware that current concepts of cholesterol and HDL physiology hinge on the whereabouts of liver SR-B1 expression, and noting that the localization of SR-B1 to the hepatocyte was not precise^6^,^7^,^8^,^17^, we were prompted to consider instead that SR-B1 is expressed in the LSEC. We therefore set out to determine exactly where in the liver SR-B1 protein resides using two fundamentally different IF strategies. First, we resolved two closely situated cell types *in situ* using a special microscopic sample preparation technique; and, second, we separated the two cell types by physical properties and then analyzed each by flow cytometry.

In preparation for our first strategy we analyzed conventional mouse liver sections (5 μm-thick) using plasma membrane marker, RIIb, and sub-plasma membrane marker, β-catenin, for LSEC and hepatocytes respectively; and then we analyzed the distribution of SR-B1 by IF confocal microscopy.

**Figure 1:**
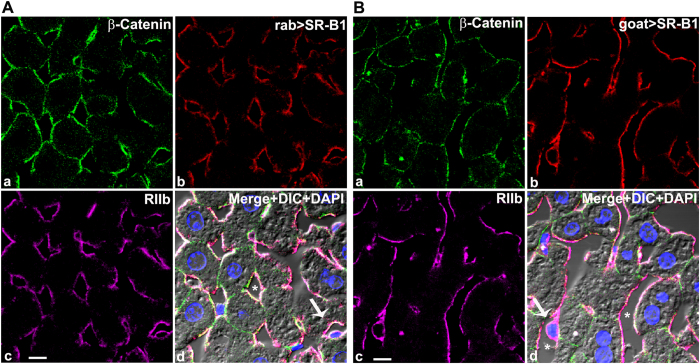
Where in liver is SR-B1 expressed? (**A**) Confocal microscopic images of a 5 μm thick mouse liver section stained with fluor-tagged mouse IgG anti-β-catenin, rabbit IgG anti-SR-B1, mab 2.4G2 IgG anti-RIIb, and DAPI. (**a**) Hepatocyte membrane marker β-catenin (green). (**b**) anti-SR-B1 antibody (red). (**c**) LSEC membrane marker RIIb (pseudo colored magenta). (**d**) The panel shows the merged color images plus DIC and DAPI staining of nuclei. Note that SR-B1 colocalizes with the sinusoidal domain of hepatocytes and throughout the LSEC. The scale bar in panel c equals 5 μm (**a**–**d**). (**B**) Confocal microscopic images of a 5 μm thick mouse liver section stained with fluor-tagged mouse rabbit IgG anti-β-catenin, goat IgG anti-SR-B1, mab 2.4G2 IgG anti-RIIb, and DAPI. (**a**) Hepatocyte membrane marker β-catenin (green). (**b**) anti-SR-B1 antibody (red). (**c**) LSEC membrane marker RIIb (pseudo colored magenta). (**d**) Merged color images plus DIC and DAPI staining of nuclei. Note that the SR-B1 colocalizes with the sinusoidal domain of hepatocytes and throughout the LSEC. The 5 μm bar in panel c applies to all panels. Arrow in panel d points to the nucleus of an LSEC circumferentially lined by both RIIb and SR-B1. In panel d * identifies the sinusoidal lumen.

The LSEC plasma membrane marker RIIb appeared uniformly expressed in liver sinusoidal endothelial cells lining the lumen giving a hallmark LSEC pattern^12^. In contrast, β-catenin was detected only in hepatocytes, at both the basal lateral (sinusoidal) and the apical (bile canalicular) surfaces (Fig. 1, top left panels) as reported earlier by others^18^,^19^,^20^. The molecule of interest, SR-B1, identified with specific antibodies, appeared in a sinusoidal pattern aligning perfectly with the LSEC marker RIIb (Fig. 1A,B, top right and bottom left panels). Further, the appearance of the SR-B1 signal on the abluminal plasma membrane of LSEC as the plasma membrane passes around the nucleus (arrows in Fig. 1A,B panel d) (seen about 10 times), suggests strongly the conclusion that the LSEC expressed SR-B1.

However, SR-B1 also appeared to be expressed on the hepatocyte surface, identified with the β-catenin marker, but only on the basal lateral surface of the hepatocyte in confocal optical sections. No binding of anti-SR-B1 appeared near the bile canaliculi (Fig. 1, top left panels).

Our observations about SR-B1 sites in liver sections were seen with all three anti-SR-B1 antibodies, viz., two polyclonal anti-peptide antibodies from rabbit and one from goat, all directed toward a cytoplasmic tail sequence of SR-B1. We found that all three anti-SR-B1 antibodies displayed similar signals in LSEC that were much brighter and more abundant than the signals seen in hepatocytes (Fig. 1A,B and data not shown).

### Validation of SR-B1 antibody specificity

**Figure 2:**
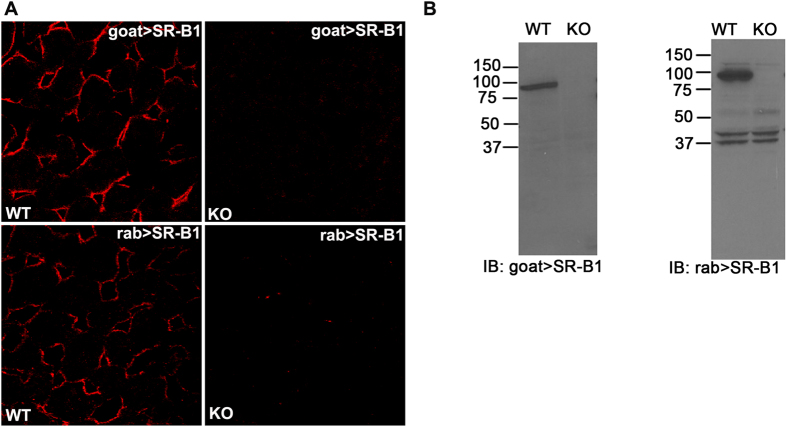
Validation of SR-B1 antibodies. (**A**) Confocal microscopic image of WT (left panels) and SR-B1 KO (right panels) liver sections labeled with goat IgG anti-SR-B1 (top row) and rabbit IgG anti-SR-B1(bottom row). (**B**) ECL immunoblots developed using goat (left) and rabbit (right) anti-SR-B1 showing the presence and absence of the SR-B1 band (molecular weight ~ 82 kDa) in WT and KO liver lysates, respectively. Numbers are MW markers in kDa. Note the presence of low MW non-specific bands of equal intensity in WT and KO, supporting equal protein loading (10 μg) in both lanes. Gel images are not cropped except for the irrelevant lateral lanes.

Antibody specificities of goat anti-SR-B1 and rabbit anti-SR-B1 (Krieger) were validated by IF confocal microscopy (Fig. 2A) and immunoblotting (IB) using liver from WT and SR-B1 KO strains. The lack of detection by anti-SR-B1 antibodies of the specific IF signal in KO liver sections (Fig. 2A) recommends both antibodies. The other rabbit anti-SR-B1 antibody (Novus) showed specificity similar to the rabbit SR-B1 (Krieger) used in Fig. 2 (data not shown). Dissimilarly, IB indicates that the goat anti-SR-B1 antibody is more specific and shows no cross-reactivity with other antigens. Both goat anti-SR-B1 and rabbit anti-SR-B1 were specific for a single band of about 82 kDa that is completely absent in KO lysates (Fig. 2B). Because the rabbit anti-SR-B1 showed non-specific binding to some lower molecular weight proteins (Fig. 2B right panel) and because of limited availability, we were lead to use the goat antibody for the rest of our studies.

### High resolution microscopy of ultrathin tissue slices shows that SR-B1 is predominantly expressed in LSEC

**Figure 3:**
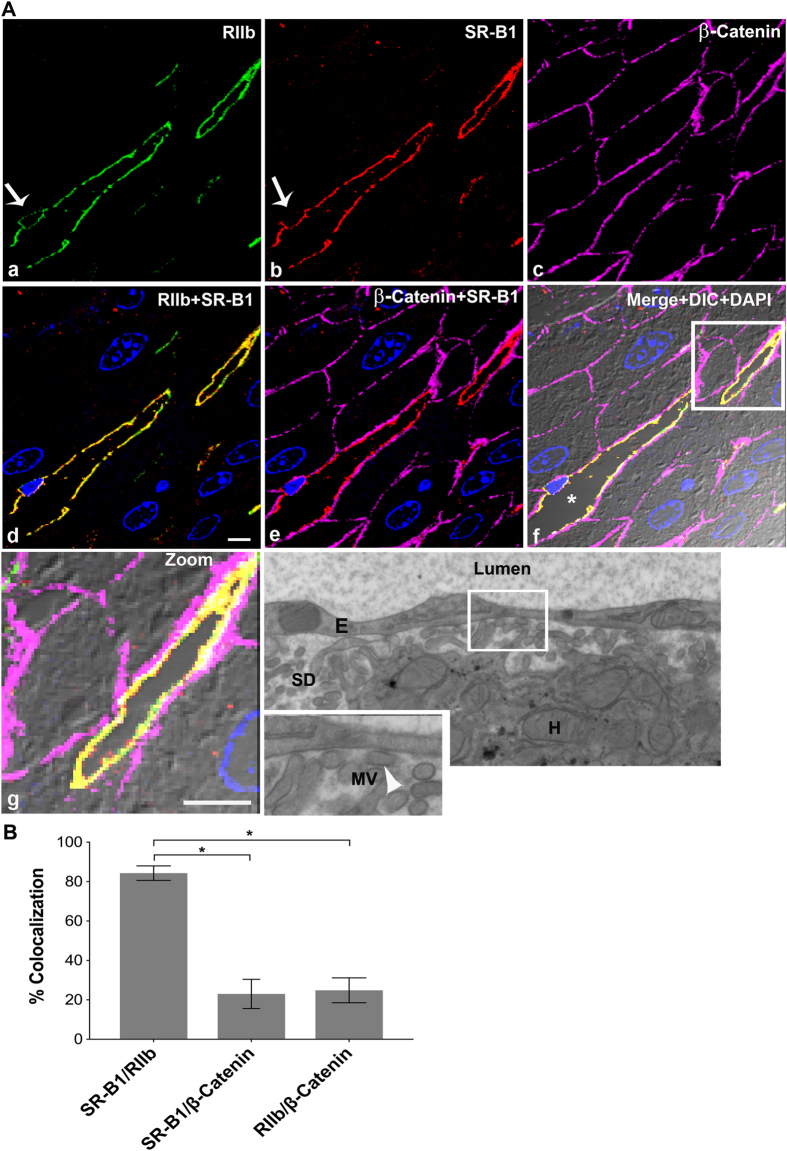
High resolution confocal image illustrates that SR-B1 is predominantly expressed in LSEC. (**A**) High-resolution 4-color immunofluorescence images of an ultrathin (120 nm) cryosection from BALB/c liver localizing mab 2.4G2 anti-RIIb (green) in panel a, goat IgG anti-SR-B1 (red) in panel b, mouse IgG anti-β-Catenin (magenta) in panel c, a merge of panels a and b in panel d, and a merge of panels b and c in panel e. Panel f shows merges of the 4 color images plus DIC and DAPI staining. Panel g shows the zoomed area outlined in panel f. Panel h shows a high magnification transmission electron micrograph of mouse liver showing the lumen of the sinusoid lined by LSEC. The Space of Disse (SD), containing numerous microvilli (MV) (arrow head) projecting from the membrane of hepatocytes (H), touching the LSEC membrane is shown in the zoomed panel of g. Arrow in panels a and b points to the nucleus of an LSEC circumferentially lined by both RIIb and SR-B1. In panel f, * identifies the sinusoidal lumen. The scale bar in panel d (a–g) equals 5 μm. (**B**) The bar graph plots the percentage of signal from anti-SR-B1 (red) colocalizing with signal from anti-RIIb (green) or anti-β-catenin (magenta) and also colocalization of both LSEC (green) and hepatocyte marker β-catenin (magenta). Using the student’s t-test, the data of bars 1 and 2 and also bars 2 and 3 were compared. *p < 0.0001.

**Figure 4:**
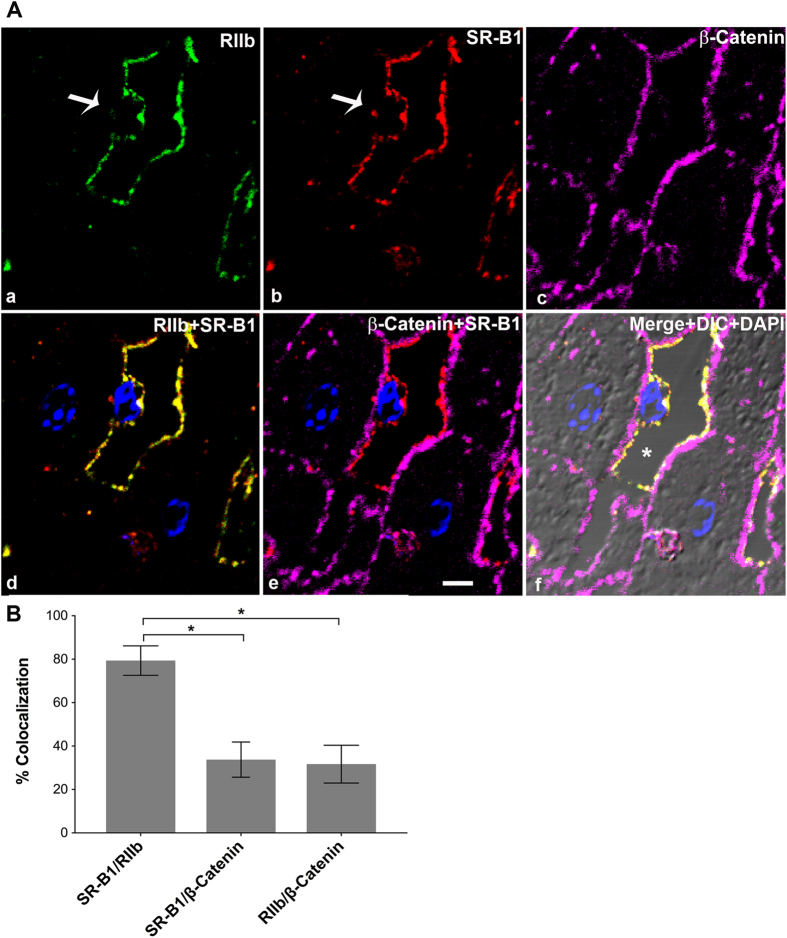
High resolution confocal imaging shows localization of SR-B1 predominantly to LSEC. (**A**) High-resolution 4-color immunofluorescence images of an ultrathin (120 nm) cryosection from C57B/6 liver localizing anti-RIIb (green) in panel a, goat IgG anti-SR-B1 (red) in panel b, anti-β-Catenin (magenta) in panel c, a merge of panels a and b in panel d, and in panel e the merge of (b,c). (f) Merge of the 4 color images plus DIC and DAPI staining. The bar in panel e indicates 5 μm (a–f). Arrow in panel a and b points to the nucleus of an LSEC circumferentially lined by RIIb and SR-B1. In panel f, * points to the lumen. (**B**) The bar graph plots the percentage of signal from anti-SR-B1 (red) colocalizing with signal from anti-RIIb (green) or with signal from anti-β-catenin (magenta) and also anti-RIIb (green) signal colocalizing with signal from anti-β-catenin (magenta). Using *student’s t-test*, the data from bars 1 and 2 and also between bar 2 and 3 were analyzed. *p < 0.0001.

In an attempt to specify the sites of SR-B1 fluorescence in closely situated LSEC and hepatocytes, we utilized a method that provides high resolution for IF localization of closely located antigens^21^. Specifically, using livers from BALB/c (Fig. 3) and C57BL/6 (Fig. 4) mouse strains, we prepared ultrathin cryosections of 120 nm thickness as substrates for labeling. In LSEC, where the nucleus is identified with DAPI (arrow) (Fig. 4), it is evident that RIIb is expressed both at the luminal and abluminal LSEC borders (Fig. 3A and 4A)^13^. In all other places where the cytoplasm is very thin both cell borders of LSEC seem merged^22^. Observe in panel d of Fig. 3 and in Fig. 4 that the staining pattern of SR-B1 is similar to the RIIb pattern, except that SR-B1 appears to be expressed more abundantly on the luminal rather than the abluminal side of LSEC. β-Catenin does not appear co-localized with RIIb in the sinusoidal plasma membranes suggesting gross resolution and separation of these two cell types. Note in the merged, zoomed panel g of Fig. 3A, a portion (the lower 1/5 of the sinusoid) of LSEC (yellow = green RIIb + red SR-B1) that has separated from the basal lateral plasma membrane of the adjacent hepatocyte (magenta β-catenin only) clearly shows that RIIb and SR-B1 are completely separated from the hepatocyte sinusoidal plasma membrane, strongly suggesting abundant expression of SR-B1 in LSEC but no apparent expression in hepatocytes. In merged 3-color IF images, SR-B1 co-localized very minimally with the basal lateral plasma membrane of hepatocytes allowing the possibility of minimal expression of SR-B1 in hepatocytes (panel e of Fig. 3 and 4).

Quantification of several images from 3 different mice, similar to Fig. 3A (Fig. 3B), showed that a majority (84% ± 4) of the SR-B1 pixels also contains the LSEC marker, while only a minority (24% ± 6) of these same SR-B1 pixels contains also the hepatocyte marker. The sum of two percentages given above, where SR-B1 pixels are shared, is close to the theoretical maximum of 100%. Repeating the same quantification shown in Fig. 3A by switching the channels reciprocally, we saw the exact same % of pixels of the LSEC marker co-localizing with SR-B1 and also of pixels of the hepatocyte marker with SR-B1 (data not shown). The reliability of the quantification method is thus assured.

To assess the capacity of our method to resolve optically two cells known to be physically separate, we colocalized RIIb with β-catenin, and saw co-localization of 25% in both directions, indicating the limitation of ultrathin sections to completely resolve the closely spaced LSEC from the sinusoidal surface of hepatocytes. Thus, we disregard apparent co-localization of 24% or less (Fig. 3B and 4B).

The electron microscopy (EM) image in Fig. 3A (panel h) showed that the microvilli of hepatocyte plasma membrane are almost touching the LSEC at several places, identified by arrow heads, within the Space of Disse identifying a limitation of fluorescence microscopy in separating the fluorescence signals emanating from the surface membranes of these two cells with the confocal imaging system employed.

To ascertain whether liver SR-B1 expression is strain or gender specific we examined a different mouse strain, female C57BL/6, and found that the two strains did not differ. We found that the distribution of SR-B1, β-catenin, and RIIb were similar in female C57BL/6 to that in male BALB/c with 79% of the SR-B1 signal in the same location as the LSEC marker RIIb (Fig. 4B) and 34% of the same SR-B1 co-localizing with β-catenin. The sum of the two percentages where SR-B1 pixel is shared (79 ± 7 and 34 ± 8) was a little over the theoretical maximum of 100%.

### Liver Non-parenchymal cells (NPC), separated from hepatocytes, express abundant SR-B1 *ex vivo*

**Figure 5:**
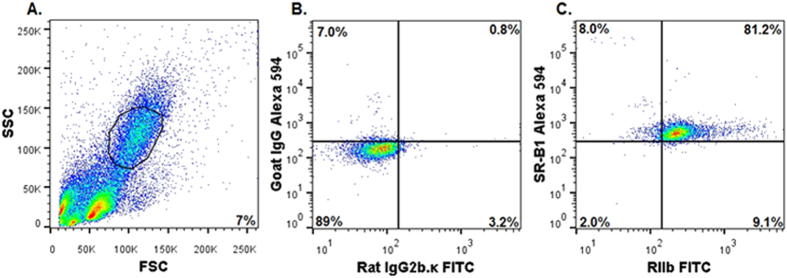
NPC of liver express abundant SR-B1, *ex vivo*. (**A**) A representative flow cytometric acquisition plot showing forward scatter (FSC) vs side scatter (SSC) of an NPC preparation, with the gate (NPC) indicated. Note the linear Y axis scale for SSC indicating smaller cells. (**B**) A two-color flow cytometric analysis of gated NPC for isotype controls FITC-rat IgG2b.κ and Alexa 594-goat IgG. (**C**) A two-color flow cytometric analysis of gated NPC using FITC-2.4G2 mab IgG anti-RIIb and Alexa 594-goat IgG anti-SR-B1. In (**A–C**) events are represented as blue points, with areas of very high density and high density colored red and yellow, respectively. The percentage of events showing single or double positive expression is indicated in respective quadrants. The results are representative of 4 different experiments.

Our first approach, microscopy, indicated that most SR-B1 was expressed in LSEC but a low expression in hepatocytes was possible. In our second approach, fundamentally different from our microscopy of ultrathin liver sections, we studied the expression of SR-B1 in freshly isolated NPC by flow cytometry. The identification of NPC was based on RIIb expression and on smaller FSC (size) and SSC (granularity) intensity values, which were plotted on a linear Y axis. The typical gating profile of NPC is a regular oval on a 30 degree slant (Fig. 5A). The events recorded above and below the gating profile, composed predominantly of Kupffer cells (positive for mab F4/80) and red blood cells, respectively, were excluded from analysis (Fig. 5A and data not shown). All RIIb and SR-B1 expression profiles were normalized to appropriate isotype controls (Fig. 5B). It can be seen that a great majority of the NPC gated population that is positive for RIIb (81%) is also positive for SR-B1 (Fig. 5C), whereas somewhat fewer RAW 264.7 cells (60%) express both receptors (Fig. S1 panel C). RAW cells co-expressing both receptors served as positive controls (Fig. S1 and S3), and COS-7 cells that show near baseline expression of both the receptors served as negative controls^23^. Also of note, very few cells express a single receptor of either kind, RIIb or SR-B1, 1% and 6%, respectively. Together these data indicate that the vast majority of RIIb-positive NPC also express SR-B1.

### Hepatocytes *ex vivo*, separated from NPC, show negligible expression of SR-B1

**Figure 6:**
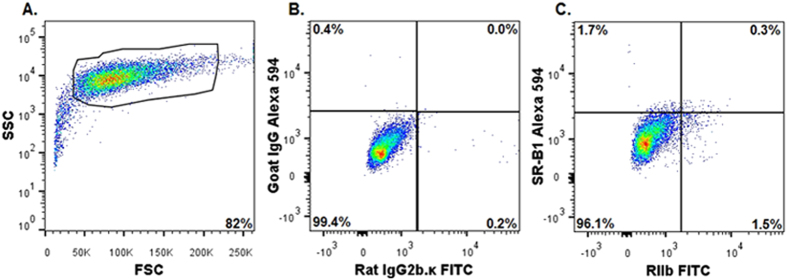
Three color flow cytometric analysis of PC of liver show negligible expression of SR-B1, *ex vivo*. (**A**) A representative flow cytometric acquisition plot showing forward scatter (FSC) vs side scatter (SSC) of a PC preparation, with the gate (PC) indicated. Note the log Y axis scale for SSC, indicating large cells. (**B**) Two-color flow cytometric analysis of gated PC for isotype controls FITC-rat IgG2b.κ and Alexa 594 goat IgG. (**C**) A two-color analysis of gated PC using FITC-mab 2.4G2 IgG anti-RIIb and Alexa 594 goat IgG anti-SR-B1. Two color flow cytometric analysis of gated PC for isotype controls Alexa 594 goat IgG and Alexa 647 mouse IgG1. (**E**) Two-color flow cytometric analysis of gated PC for Alexa 647 anti β-Catenin and Alexa 594 goat IgG anti-SR-B1. In (**A–E)**, events are represented as blue points, with areas of very high density and high density colored red and yellow, respectively. As in Fig. 5, the percentage of events showing single or double positive expression is indicated in respective quadrants. The results are representative of 3 different experiments.

A typical gating profile by flow cytometry of freshly isolated hepatocytes shows the cells to be RIIb-negative, SSC-high, and FSC-high, all of which parameters are recorded within a logarithmic Y axis scale and with instrument settings described in materials and methods^24^ (Fig. 6A). Hepatocyte cell preparations routinely yield 80–93% of cells with the phenotype described above; gating excludes RIIb-expressing NPC-contamination and cell debris, about 7–20%. It is noteworthy that 96% of the gated hepatocytes, when compared to respective isotype controls (Fig. 6B), were double negative for RIIb and SR-B1 expression, consistent with our microscopic impression in Fig. 3 and 4 (Fig. 6C). Parallel two-color analysis of the same gated hepatocytes shows that 97% of the cells were single positive for the hepatocyte marker and less than 5% were either single SR-B1 positive or double positive for SR-B1 and β-Catenin (Fig. 6E). Supplemental Fig. 4 ascertains the purity of the hepatocytes using two color flow analysis in 2 different combinations. These results indicate that hepatocytes have near nil SR-B1 expression and were similar to COS-7 cells (Fig. S2 and S3). In addition single color histogram analysis confirms the lack of SR-B1 in hepatocytes compared to hepatocyte markers, namely CD95 and β-Catenin (Fig. S5).

**Figure 7:**
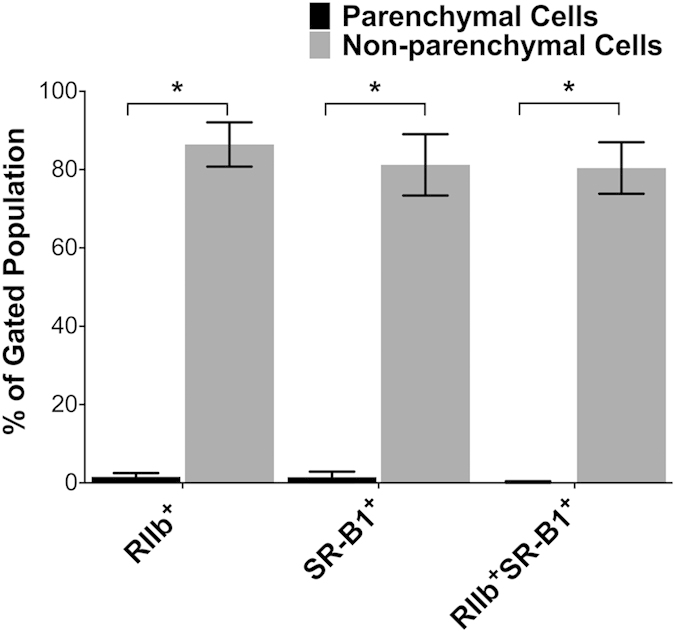
In liver, SR-B1 is expressed mostly in NPC rather than PC. Shown is a bar graph comparison of SR-B1-expression by PC and NPC: The multi-color flow-cytometry experiments described and displayed as in Fig. 5 and 6 were each repeated 4 times. The events indicated in the upper right quadrant of the 4 experiments were averaged for each cell-type and expressed in the bar-graph as mean ± SD after correcting for (subtracting) the intensity of isotype-control antibodies. *p < 0.0001.

In liver, SR-B1 is expressed considerably more abundantly in NPC than in hepatocytes. Using images such as those in Fig. 5 and 6 and S1 and S2 from 4 different mouse livers, we quantified the percent of gated hepatocytes and gated NPC that were positive for SR-B1 (Fig. 7). We found that nearly 90% of the NPC showed SR-B1 expression while the number of SR-B1 expressing cells in the gated hepatocyte population was barely perceptible.

### NPC express relatively higher SR-B1 mRNA expression per cell than hepatocytes

**Figure 8:**
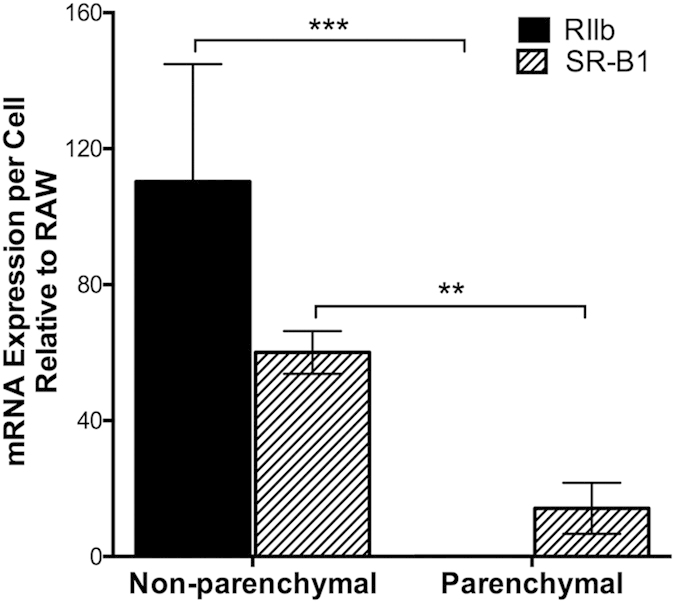
In liver, SR-B1 mRNA expression is markedly higher in NPC than PC. Freshly isolated NPC and PC preparations were sorted, and mRNA expression was analyzed using reverse transcriptase quantitative real-time PCR. Relative fluorescence units (RFU) were taken as a quantitative measure of mRNA expression. mRNA expression for both NPC and PC were displayed in a bar graph as mean ± SD mRNA expression per cell after normalization to RAW 264.7 cell RIIb and SR-B1. Statistical significance was analyzed with *student’s t-test* analysis, **p < 0.01 ***p < 0.05.

We assessed SR-B1 mRNA expression in purified NPC and hepatocytes by real time PCR and found that NPC express considerably more (~5 × more) than hepatocytes (Fig. 8). The absence of RIIb mRNA expression in hepatocytes suggests no contamination of NPC within our hepatocyte preparation.

## Discussion

We conclude from our data that liver sinusoidal endothelial cells express substantially higher SR-B1 than hepatocytes. While most of our data come from IF experiments, we have used two disparate strategies to separate the closely positioned cells of interest, LSEC and the hepatocytes. The first strategy, confocal IF microscopy of mouse liver sections, leaves the two cell types in their living state and utilizes special microscopic techniques to resolve the sources of fluorescence signals. Cell-type specific antibodies were used to identify the two cell types in sections, anti-FcγRIIb mab for LSEC and anti-β-catenin and anti-CD95 for hepatocytes.

The second strategy, flow cytometric analysis using dissociated liver cells that were incubated with fluor-tagged anti-SR-B1 antibody, separated the two cells of interest by first enzymatic dispersion of liver contents and then differential centrifugation of cell suspensions to achieve relative cell-type purity.

**Figure 9:**
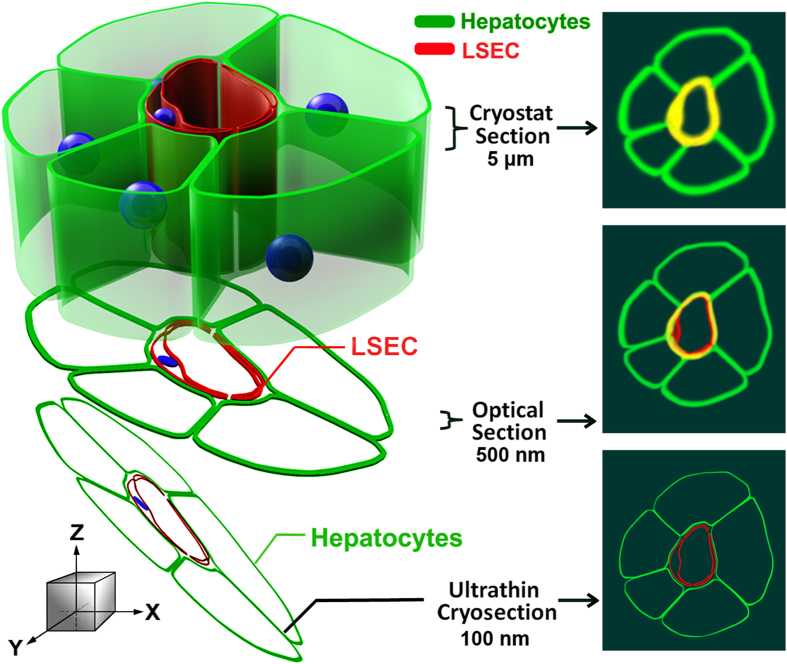
Model illustrating the advantages of using ultrathin cryosections for high-resolution IF microscopy. The figure illustrates plasma membrane structures of LSEC and hepatocytes labeled with fluor-tagged mab 2.4G2 (red) and anti-β-catenin antibody (green). (Left) Schematic model of immunostained liver sinusoid cut into sections of different thicknesses (cryostat section, optical section, and ultrathin cryosection). (Right) Fluorescence signals in the x- and y-dimensions (top view in the z direction). In a top view of conventional cryostat sections (>5 μm thick), 2.4G2 and β-catenin fluorescence signals are indistinguishable, thus presenting false co-localization (yellow). In a confocal view of optical sections (~500 nm thick), the potential for false co-localization is reduced due to less signal diffusion compared to thicker cryostat sections. However, a detailed relationship of LSEC and hepatocytes is still difficult to resolve due to blurring of 2.4G2 and β-catenin fluorescence signals. In a confocal view of ultrathin cryosections (50–150 nm thick), the signal diffusion of fluorescence signals is minimized and the two cells are distinct.

The critical feature of our microscopic strategy was the use of ultrathin cryosections of liver tissue that enabled us to distinguish fluorescence signals from hepatocytes and LSEC that were not distinguishable with confocal microscopy of 5 μm sections. The improved resolution obtained with ultrathin sections compared to optical sections of thicker sections is largely due to improved resolution in the z-axis. In a confocal microscope, the z-axis resolution is limited by diffraction to ~700 nm. It has been estimated that the imaging of ultrathin sections may lead to a 5–7-fold improvement in z-resolution^25^ and can be comparable to super resolution microscopy. One of us (JMR) who pioneered this approach has pointed out earlier that the use of ultrathin sections minimizes the false co-localization that is more likely to occur with lower resolution images^21^,^26^,^27^,^28^. We illustrate the major advantages of ultrathin cryo sections in a cartoon (Fig. 9) that demonstrates how false co-localization is minimized.

Using ultrathin sections we determined that the great majority of the IF signal of SR-B1 co-localized with the fluor-tagged marker for LSEC. Most of the SR-B1 was expressed by LSEC, regardless of the genetic background/gender of the mice.

Evaluation of hepatocyte expression of SR-B1 viewed in ultrathin sections was more complex. We saw a low intensity fluorescence signal of SR-B1 co-localized with hepatocytes identified with the β-catenin marker. However, the LSEC marker, RIIb, also co-localized with hepatocytes and gave the same percentage of co-localization with the β-catenin marker. Thus we cannot eliminate the possibility that a small amount of SR-B1 is expressed in the hepatocytes near the sinusoidal face, but we are prevented by the optical limitations of our microscopy system from being more definite and precise. However, using our second strategy, flow cytometric analysis of dissociated liver cells, we were able to be more definitive about SR-B1 expression. Separating the dissociated liver cells into two pools, hepatocytes and non-parenchymal cells (NPC, chiefly LSEC), we noted that virtually the entire SR-B1 signal emanated from NPC, and nearly nil from the hepatocytes.

Thus, we would conclude from our two physically different strategies that SR-B1 is undeniably expressed in considerable abundance by LSEC while hepatocytes express near none. Why is our conclusion so obviously different from what has appeared in the literature so far? It is our impression that as far back as the first localization of tissue SR-B1 there were technical limitations to the approaches employed. From studies of purified membranes from the entire liver, which contains many types of cells, it was suggested that SR-B1 was expressed only in hepatocytes^7^. A number of laboratories supported this finding using approaches with technical limitations^6^,^8^,^17^. From transfection studies it was presumed that SR-B1 was found in specific areas of the hepatocyte, but the possibility of ectopic expression was not eliminated nor was the site of SR-B1 expression under native conditions pursued^16^. It is easy now to understand why cultured hepatocytes failed to show SR-B1 expression, for hepatocytes very weakly express SR-B1 and those abundant SR-B1 expressing cells that might have contaminated the hepatocyte preparation, namely LSEC, are known to lose at least one marker (FcγRIIb) in culture^29^. It would not be surprising for them to lose another surface protein, SR-B1^11^. Additionally, contaminating LSEC may not persist under conditions favoring hepatocyte culture.

Our study also shows that mRNA for SR-B1 per cell is relatively more abundant in LSEC than hepatocytes by a factor of about five. These results differ from those of others who have studied liver cells from mouse and rat that are pure only by morphologic criteria^30^,^31^. Given the presence of messenger, why we saw so little protein expression of SR-B1 in hepatocytes is not obvious. A variety of possibilities occur to us; e.g., post-transcriptional regulation^32^, narrow antibody specificity, and others.

Regarding the expression of SR-B1 in other cells of the liver, it is also possible that the Kupffer cells of liver express SR-B1. We did not see labeling of SR-B1 around the bile duct using anti-SR-B1 antibodies in liver IF, suggesting that the cholangiocytes may not have detectable amounts of SR-B1. We acknowledge that stellate cells may express SR-B1, but expression should be negligible as reported earlier^33^. The expression of SR-B1 in other cells of the liver requires more study.

If, as we suggest, hepatocytes express little SR-B1, how does cholesterol as HDL-C enter hepatocytes to be metabolized and eventually secreted into the bile? There are several issues to consider. First, it is conceivable that a small amount of SR-B1 that we cannot easily visualize is present in hepatocytes. Second, is HDL-C processed by the SR-B1-bearing LSEC such that it is made available to the hepatocyte? We know of no data to address this possibility. However, one might posit a transcytosis mechanism which involves first endocytosis, then transport across LSEC, and last exocytosis of intact HDL-C by LSEC SR-B1. One would posit that HDL-C present in the Space of Disse would then be handled by an SR-B1 or an unknown HDL receptor in hepatocytes^5^. Similar trancytosis mechanisms mediated by SR-B1 have been demonstrated in endothelial cells of lymph nodes and aorta^34^,^35^.

Another possible mechanism describes cholesterol, transcytosed by SR-B1 across LSEC, being loaded onto ApoA1 or HDL which have been synthesized by hepatocytes and are present in the Space of Disse. However, we find no claims of the presence of HDL, ApoA1, or any other form of cholesterol in the Space of Disse. These studies must be done. Next, one must consider that other HDL receptors found in hepatocytes may mediate the uptake of HDL-C in the Space of Disse after exocytosis by LSEC. In this regard, CD36^36^, the ectopic β chain of ATP synthase^37^, and an uncharacterized receptor^6^ have been studied. There may be additional mechanisms to consider.

Our compelling evidence of abundant SR-B1 expression in LSEC may stimulate new work leading to the replacement of current concepts of how cholesterol enters hepatocytes.

## Methods

All methods carried out in this manuscript were approved by the Institutional Biosafety Committee of The Ohio State University. All experiments were executed in accordance with the approved methods and guidelines.

### Animals

Wild type male BALB/c and C57BL/6 mice of age 12–15 weeks were obtained from Jackson Laboratory. The Ohio State University Institutional Animal Care and Use Committee approved all animal studies; all experiments were executed in accordance with the approved guidelines. The SR-B1 KO and its WT strain (C57BL/6) were obtained from Dr. Monty Krieger, MIT, Cambridge, MA; and Dr. Bernardo Trigatti, McMaster University, Hamilton, Ontario, Canada.

### Cells

RAW 264.7 and COS-7 cells were obtained from The American Type Culture Collection. COS-7 cells were maintained in Dulbecco’s modified Eagle’s medium supplemented with 10% fetal bovine serum. RAW 264.7 were maintained in RPMI 1640 medium supplemented with 10% fetal bovine serum.

### Immunofluorescence

Small pieces of mouse livers (~5 mm) were fixed in 4% paraformaldehyde-PBS for 2 hrs at room temperature. Tissue was washed with PBS and infused with 20% sucrose-PBS overnight at 4 °C. The tissue was then embedded in a tissue freezing medium for cryostat sectioning and stored at −80 °C until used. Cryostat sections, 5 μm thickness, were collected on Superfrost microscope slides (Fisher Scientific). The sections were blocked in 5% milk-PBS prior to incubation with primary antibodies overnight at 4 °C. The primary antibodies used in the study were Alexa 647/488 fluor-tagged mouse IgG anti-β-catenin, rabbit polyclonal anti-SR-B1 C-terminus (gift from Dr. Krieger), rabbit polyclonal C-terminal anti-peptide mouse SR-B1 (450–509)(Novus Biologicals), goat polyclonal anti-C-terminal peptide of mouse SR-B1 (450–509) (Novus Biologicals), mab 2.4G2 IgG anti-RIIb (BD Biosciences).

Unconjugated primary antibody binding was localized using fluorophore-conjugated secondary antibodies in blocking buffer for 1 hr at 4 °C. Secondary antibodies used for this studies were goat IgG anti-rat IgG (conjugated with dyes Alexa 647, 680 or 488), and goat IgG anti-rabbit IgG (conjugated with Alexa dyes 647, 488 or 568) from Life technologies. The Cy3 AffiniPure F(ab′)2 fragment donkey anti-goat IgG was bought from Jackson Immuno Research. Unless specified all secondary antibodies were used at a 1:200 dilution. Nuclei were stained with DAPI for 10 minutes and the sections were mounted under cover slips in Prolong Gold Anti-Fade (Life Technologies). Control incubations included isotype controls, respective secondary antibodies, and secondary antibodies alone.

In Fig. 3 and 4, to overcome the inherent limits of resolution of two close antigens in the z-dimension in our confocal microscopes, we used ultrathin cryosections of liver tissue of 120 nm thickness compared to regular 5 μm thick sections as the substratum for high-resolution IF microscopy as we have described^27^,^28^,^38^. Preparation of tissue and ultrathin cryosections we described in detail earlier^39^.

Both the 5 μm and ultrathin sections were examined and images were acquired in the Olympus Fluo View 1000 Laser Scanning Confocal microscope equipped with a spectral detection system for a finer separation of fluorochromes (FV 1000 Spectra). Images were acquired with the FluoView software system.

### Quantification of IF images

The fluorescence intensities from RIIb (green) and SR-B1 (red) and β-catenin (magenta) channels in the ultra-thin sections represented in Fig. 3 and 4 were obtained using Olympus FV1000-ASW analysis software. IF from RIIb/SR-B1, SR-B1/β-catenin and RIIb/β-catenin was co-localized in 28 IF optical sections, totaling 1.2 and 1.9 mm^2^ from 3 mice each for BALB/c and C57BL/6, respectively. Co-localization analysis was performed using Olympus FV1000-ASW analysis software (Ver. 3.1). The Mander’s overlap coefficient is expressed as percent colocalization.

### Immunoblot

Liver lysates from WT and SR-B1 KO mice were prepared as described^13^. The proteins were separated on 10% SDS-polyacrylamide gels and were transferred to nitrocellulose membranes (0.45 μm). The membranes were blocked with 5% milk for 30 minutes and then incubated overnight with primary rabbit anti-SR-B1 antibody (kind gift from Dr. Monty Krieger) and goat IgG anti-SR-B1 (Novus Biologicals) antibodies at concentrations of 1:5000 and 1:500, respectively, at 4 °C. The bands were developed using HRP conjugated goat anti-rabbit IgG antibody at a concentration of 1:1000 and ECL developed.

### Isolation and purification of PC

Hepatocyte isolation and purification was performed as described previously^40^,^41^. In brief, the liver was perfused with 0.09% EGTA-containing calcium-free salt solution followed by a 0.05% collagenase solution (type IV, Sigma, St. Louis, MO) in 1% albumin, through the portal vein. The digested liver tissue was minced, filtered, and washed in RPMI-1640 with 10% FBS. Hepatocytes were purified on a 50% Percoll gradient (Pharmacia Biotech).

### Isolation and purification of NPC

The NPC were isolated using a modified protocol as described previously^42^. Briefly, the mouse liver was perfused via the portal vein with a perfusion buffer containing NaCl (0.14 M), KCl (6.7 mM) and HEPES (1 mM) pH 7.4, followed by Liberase TM (Roche) containing perfusion buffer at 37 °C. The digested liver containing isolated cells was excised and Glisson’s capsule was removed. The tissue debris was filtered and was followed by centrifugation at 60 × g thrice at 4 °C to remove hepatocyte pellet. The supernatants were centrifuged at 1350 × g at 4 °C to obtain a cell pellet. The pellet was resuspended in perfusion buffer containing 1% BSA and separated on a Percoll (Sigma-Aldrich) density gradient (25%/50%). The cells in the interface between 25% and 50% were collected and washed further with perfusion buffer containing 1% BSA to obtain non-parenchymal cells. NPC were identified in flow sorting with directly labelled antibodies against 2.4G2 and F4/80. The red blood cell contamination and cell debris were excluded based on the lack of staining with the above antibodies.

### Alexa 594 labeling of goat IgG anti-SR-B1 antibody

The goat IgG isotype control and the goat IgG anti-SR-B1 were labeled with Alexa Fluor 594 using succinimidyl ester of Alexa Fluor 594 (Life Technologies) according to the manufacturer’s instructions.

### Multi-color flow cytometry sample preparation

The PC (immediately after isolation), RAW 264.7 and COS-7 cells were blocked using human IgG at 2 mg/ml (Sigma). After blocking, cells were stained with FITC-mab 2.4G2 IgG anti-RIIb and PE anti-CD95 or FITC-rat IgG2b.κ and PE Hamster IgG2, λ1 for 30 minutes at 4 °C. Stained and washed cells were then fixed with 4% paraformaldehyde at room temperature, followed by additional washing. The cells were then permeabilized using 0.1% Triton X 100 for 15 minutes at room temperature. The permeabilized cells were labelled with Alexa 594-goat IgG anti-SR-B1 and Alexa 647 anti-β-Catenin or Alexa 594-goat IgG and mouse IgG1 in human IgG, primary and isotype controls, respectively. After two washes with PBS-FACS, the cells were analyzed by flow cytometry. The NPC were processed similarly, except the cells were fixed immediately after isolation and permeabilized using ice cold methanol and immediately diluted with PBS-FACS.

### Multi-color analysis by flow cytometry

Two and three color flow cytometry analysis was performed using BD FACS Aria III Flow Cytometer equipped with 4 lasers. Analyses were performed using FlowJo software. The instrument setting for hepatocytes includes removing the flow line filters that were set to exclude aggregates of smaller cells and changing the voltage to accommodate larger cells. The hepatocytes gates were set based on the size and confirmed by the lack of FcγRII/III staining as shown by *Gonçalves et al.* 2007^24^. The NPC gating strategy was optimized to exclude contaminating events, particularly RBC and cellular debris. To avoid spectral overlap, compensations were done by subtracting unwanted signals using single and double stained control samples. The specificities of the flow antibodies were confirmed using RAW 264.7 (FcγRII/III+ and SR-B1+) and COS-7(FcγRII/III- and SR-B1-) cell lines. The quadrant markers were set based on the isotype controls. For each analysis 10,000 events were analyzed.

### Transmission electron microscopy

Electron microscopy sample preparation was based on our previous method^43^ and sections were then examined using a FEI Tecnai G2 Spirit Transmission Electron Microscope (TEM). LSEC were identified as very thin cells, thick at the nuclei, defining the sinusoidal lumens, overlying the Space of Disse. Hepatocytes were identified as very large cells, containing abundant mitochondria and with microvilli projecting into the Space of Disse beneath the LSEC.

### Flow cytometric sorting and real time PCR analysis

NPC and PC preparations (immediately after isolation) were stained with FITC-mab 2.4G2 IgG anti-RIIb and PE anti-CD95 or FITC-rat IgG2b.κ and PE Hamster IgG2, λ1 for 30 minutes at 4 °C. Stained cells were washed and suspended in FACS sorting buffer (1x PBS, 1 mM EDTA, 25 mM HEPES pH 7, 1% FBS). Sorting of NPC and PC preparations was performed using a BD FACS Aria III Flow Cytometer equipped with 4 lasers. Similar to flow cytometric analysis, PC sorting required changing of the filter to accommodate the larger cells. Sorted NPC (FcγRII/III + ) and PC (FcγRII/III-CD95+) were stored in RPMI recovery media (20% FBS). Sorted NPC, sorted PC, RAW 264.7, and COS-7 cells were lysed in TRIzol and total RNA was isolated with Qiagen RNAeasy columns. Superscript III reverse transcriptase enzyme (Invitrogen) was used to reverse transcribe RNA to cDNA. Real time PCR was performed using a SR-B1 and FcγRII/III TaqMan gene primers (Applied Biosystems) and 1 μg cDNA. The mRNA expression per cell was determined using the relative fluorescent units (RFU), total RNA isolated, and number of cells lysed. NPC and PC expression levels were normalized to RAW 264.7 cells, positive control. Triplicate samples were analyzed in duplicate wells in each experiment.

## Additional Information

**How to cite this article**: Ganesan, L. P. *et al.* Scavenger receptor B1, the HDL receptor, is expressed abundantly in liver sinusoidal endothelial cells. *Sci. Rep.* **6**, 20646; doi: 10.1038/srep20646 (2016).

## Supplementary Material

Supplementary Information
