# Supplementary material for: Scavenger receptor B1, the HDL receptor, is expressed abundantly in liver sinusoidal endothelial cells
Source: Sci Rep. 2016 Feb 11;6:20646. doi: 10.1038/srep20646 (PMC4749959; doi:10.1038/srep20646)
Supplement: Supplementary Information [file srep20646-s1.doc]

**TITLE**: Scavenger receptor B1, the HDL receptor, is expressed abundantly in liver sinusoidal endothelial cells.

**AUTHORS**: Latha P. Ganesan^1*^, Jessica M. Mates^1^, Alana M. Cheplowitz^1^, Christina L. Avila^2^, Jason M. Zimmerer^2^, Zhili Yao^1^, Andrei Maiseyeu^3^, Murugesan V. S. Rajaram^4^, John M. Robinson^5^, and Clark L. Anderson^1*^

**AFFILIATIONS**: ^1^Department of Internal Medicine, ^2^Department of Surgery, ^4^Department of Microbial Infection and Immunity, ^5^Department of Physiology and Cell Biology, The Ohio State University, Columbus, OH 43210. ^3^Department of Medicine, Division of Cardiovascular Medicine, University of Maryland, Baltimore, MD 21201. *corresponding authors

**CORRESPONDING AUTHORS**:

Dr. Latha P. Ganesan and Dr. Clark L. Anderson, Dept. of Internal Medicine, The Ohio State University, 392 Biomedical Research Tower, 460 West Twelfth Avenue, Columbus, OH 43210. Phone: (614) 247-7650; Fax: (614) 247-7669; email: [Ganesan.Prabha@osumc.edu](mailto:Ganesan.Prabha@osumc.edu) and [anderson.48@osu.edu](mailto:anderson.48@osu.edu)

**RUNNING HEAD**: SR-B1 is expressed in liver sinusoidal endothelium

**Figure S1.**


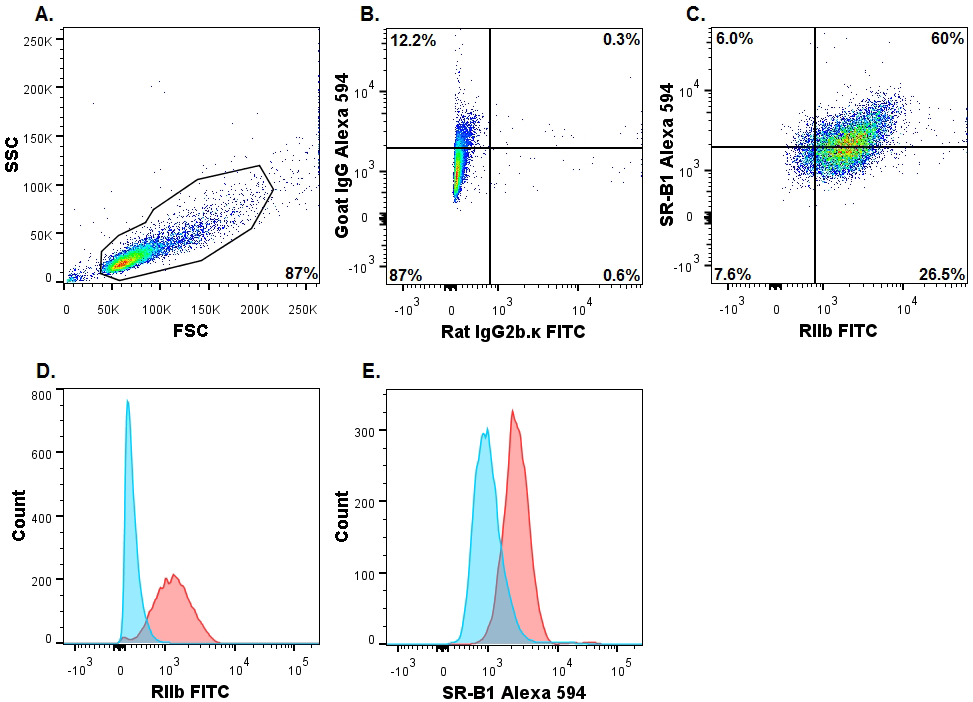


**Figure S1. RAW 264.7 cells express RIIb and SR-B1. A.** A representative flow cytometric acquisition plot showing forward scatter (FSC) vs side scatter (SSC) of RAW 264.7 cells, with the gate indicated. **B.** Two-color flow cytometric analysis of gated RAW 264.7 for FITC-rat IgG2b.κ and Alexa 594 goat IgG. **C.** Two-color analysis of gated RAW 264.7 using FITC-mab 2.4G2 IgG anti-RIIb and Alexa 594 goat IgG anti-SR-B1. In A-C, events are represented as blue points, with areas of very high density and high density colored red and yellow, respectively. The percentage of events showing single or double positive expression is indicated in respective quadrants. **D.** Flow cytometry histogram of RAW 264.7 cells stained with FITC-rat IgG2b (blue) and FITC-mab 2.4G2 IgG (red). **E.** Representative flow cytometry histogram of RAW 264.7 cells stained with Alexa 594 goat IgG (blue) and Alexa 594 goat IgG anti-SR-B1 (red).

**Figure S2.**


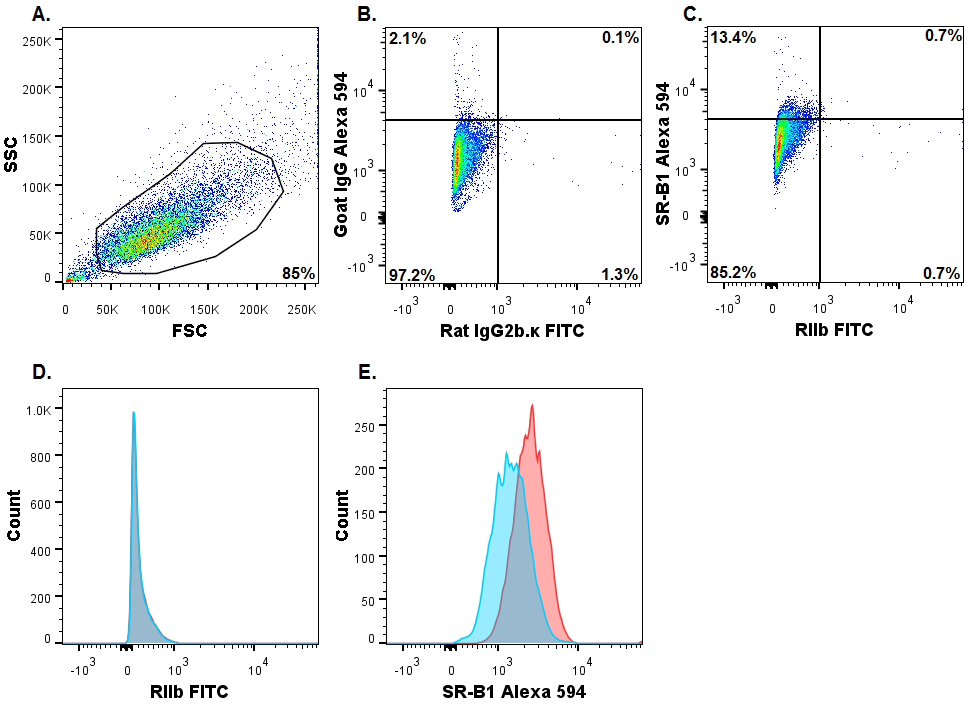


**Figure S2. COS-7 cells do not express RIIb and express SR-B1 weakly. A.** A representative flow cytometric acquisition plot showing forward scatter (FSC) vs side scatter (SSC) of a COS-7 cells, with the gate indicated. **B.** Two-color flow cytometric analysis of gated COS-7 cells with isotype controls FITC-rat IgG2b.κ and Alexa 594 goat IgG. **C.** Two-color analysis of gated COS-7 cells using FITC-mab 2.4G2 IgG anti-RIIb and Alexa 594 goat IgG anti-SR-B1. In A-C, events are represented as blue points, with areas of very high density and high density colored red and yellow, respectively. The percentage of events showing single or double positive expression is indicated in respective quadrants. **D.** Flow cytometry histogram of COS-7 cells stained with FITC-rat IgG2b (blue) and FITC-mab 2.4G2 IgG (red). **E.** Representative flow cytometry histogram of RAW 264.7 cells stained with Alexa 594 goat IgG (blue) and Alexa 594 goat IgG anti-SR-B1 (red).

**Figure S3**


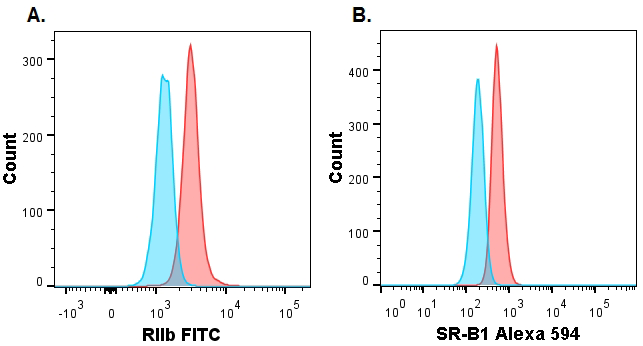


**Figure S3. NPC express both RIIb and SR-B1. A.** Flow cytometry histogram of freshly-isolated NPC stained with FITC-rat IgG2b (blue) and FITC-mab 2.4G2 IgG (red). **B.** Flow cytometry histogram of NPC stained with Alexa 594 goat IgG (blue and Alexa 594 goat IgG anti-SR-B1 (red). The results are representative of 3 different experiments.

**Figure S4**


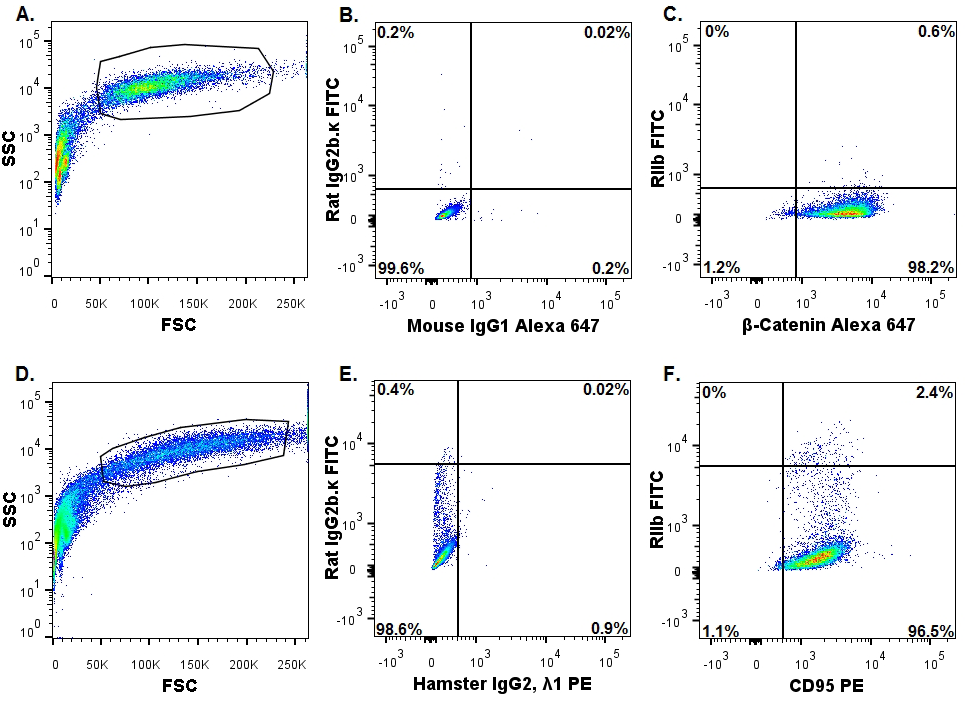


**Figure S4. Hepatocytes express β-Catenin and CD95, but do not express RIIb.**

**A.** A representative flow cytometric acquisition plot showing forward scatter (FSC) vs side scatter (SSC) of a PC preparation, with the gate (PC) indicated. **B.** Two-color flow cytometric analysis of gated PC for FITC-rat IgG2b.κ and Alexa 647 Mouse IgG1 **C.** Two-color analysis of gated PC using FITC-mab 2.4G2 IgG anti-RIIb and Alexa 647 anti-β-Catenin **D.** A representative flow cytometric acquisition plot showing forward scatter (FSC) vs side scatter (SSC) of a PC preparation, with the gate (PC) indicated. **E.** Two-color flow cytometric analysis of gated PC for FITC-rat IgG2bκ and PE Hamster IgG2λ1. **F.** Two-color flow cytometric analysis of gated PC for FITC-mab 2.4G2 IgG anti RIIb and PE anti CD95. In A-F, events are represented as blue points, with areas of very high density and high density colored red and yellow, respectively. The percentage of events showing single or double positive expression is indicated in respective quadrants. The results are representative of 3 different experiments.

**Figure S5**


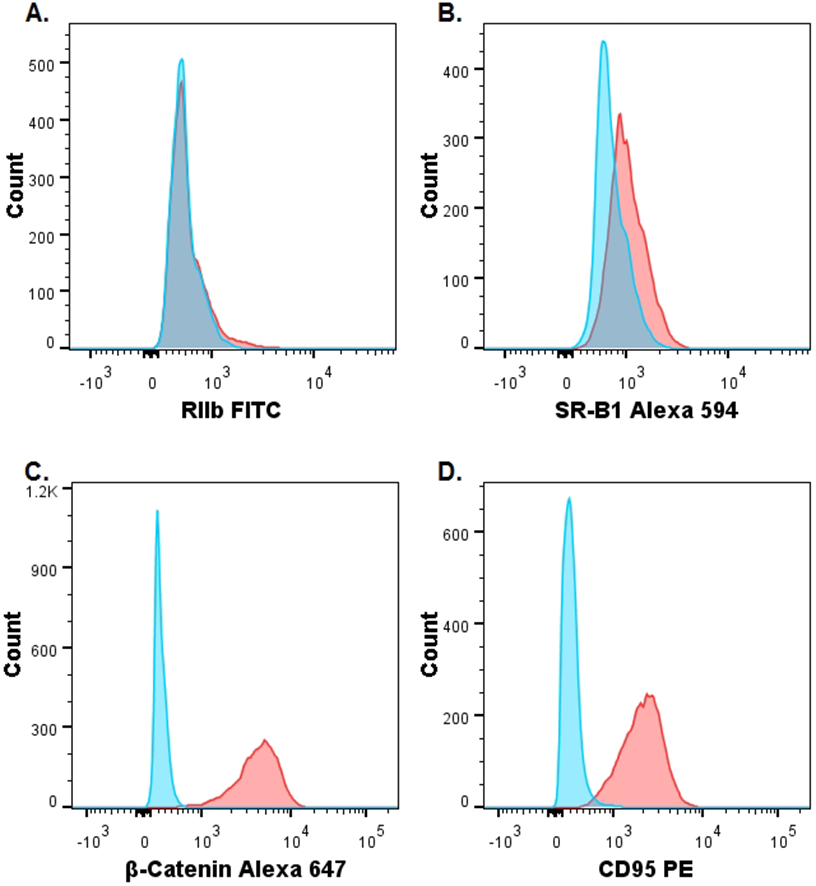


**Figure S5**. **Hepatocytes express near nil SR-B1, but express abundant β-Catenin and CD95.**

**A**. Flow cytometry histogram of freshly-isolated hepatocytes stained with FITC-rat IgG2b (blue) and FITC-mab 2.4G2 IgG (red). **B**. Flow cytometry histogram of hepatocytes stained with Alexa 594 goat IgG (blue) and Alexa 594 goat IgG anti-SR-B1 (red). **C.** Flow cytometry histogram of hepatocytes stained with Alexa 647 mouse IgG1 (blue) and Alexa 647 anti-β-Catenin (red). **D**. Flow cytometry histogram of hepatocytes stained with PE Hamster IgG2λ1 (blue) and PE anti-CD95 (red). The results are representative of 3 different experiments.
